# Neuroprotective Potential of Pituitary Adenylate Cyclase Activating Polypeptide in Retinal Degenerations of Metabolic Origin

**DOI:** 10.3389/fnins.2019.01031

**Published:** 2019-10-09

**Authors:** Robert Gábriel, Etelka Pöstyéni, Viktória Dénes

**Affiliations:** ^1^Department of Experimental Zoology and Neurobiology, University of Pécs, Pécs, Hungary; ^2^János Szentágothai Research Centre, University of Pécs, Pécs, Hungary

**Keywords:** PACAP, signaling, retina degeneration, metabolic origin, neuroprotection

## Abstract

Pituitary adenylate cyclase-activating polypeptide (PACAP1-38) is a highly conserved member of the secretin/glucagon/VIP family. The repressive effect of PACAP1-38 on the apoptotic machinery has been an area of active research conferring a significant neuroprotective potential onto this peptide. A remarkable number of studies suggest its importance in the etiology of neurodegenerative disorders, particularly in relation to retinal metabolic disorders. In our review, we provide short descriptions of various pathological conditions (diabetic retinopathy, excitotoxic retinal injury and ischemic retinal lesion) in which the remedial effect of PACAP has been well demonstrated in various animal models. Of all the pathological conditions, diabetic retinopathy seems to be the most intriguing as it develops in 75% of patients with type 1 and 50% of patients with type 2 diabetes, with concomitant progression to legal blindness in about 5%. Several animal models have been developed in recent years to study retinal degenerations and out of these glaucoma and age-related retina degeneration models bear human recapitulations. PACAP neuroprotection is thought to operate through enhanced cAMP production upon binding to PAC1-R. However, the underlying signaling network that leads to neuroprotection is not fully understood. We observed that (i) PACAP is not equally efficient in the above conditions; (ii) in some cases more than one signaling pathways are activated; (iii) the coupling of PAC1-R and signaling is stage dependent; and (iv) PAC1-R is not the only receptor that must be considered to interpret the effects in our experiments. These observations point to a complex signaling mechanism, that involves alternative routes besides the classical cAMP/protein kinase A pathway to evoke the outstanding neuroprotective action. Consequently, the possible contribution of the other two main receptors (VPAC1-R and VPAC2-R) will also be discussed. Finally, the potential medical use of PACAP in some retinal and ocular disorders will also be reviewed. By taking advantage of, low-cost synthesis technologies today, PACAP may serve as an alternative to the expensive treatment modelities currently available in ocular or retinal conditions.

## Introduction

Neuropeptides have a fundamental role in the maturation of the nervous system and their functional consequences appear in countless biological mechanisms, both in physiological and in pathological conditions. Peptides may act as neurotransmitters, neuromodulators or neurohormones, therefore their function in neuronal development/regeneration may confer crucial protective roles during pathological conditions (Strand, 2003; Casini, 2005; Cervia and Casini, 2013).

Pituitary adenylate cyclase-activating polypeptide (PACAP) was first isolated from ovine hypothalamic extract as a 38 amino acid long peptide (PACAP1-38) in 1989 (Miyata et al., 1989). It belongs to the vasoactive intestinal peptide (VIP)/secretin/glucagon peptide family members and has another isoform eleven amino acids shorter (PACAP1-27) which is less dominant in vertebrates (Arimura and Shioda, 1995; Vaudry et al., 2009). Unless stated otherwise, we refer PACAP1-38 as PACAP throughout this paper.

The biological effects of PACAP are mediated by three types of G-protein coupled receptors which have seven transmembrane domains (PAC1-R, VPAC1-R, VPAC2-R, see below). PACAP binds to pituitary adenylate cyclase-activating polypeptide type I receptor (PAC1-R) with approximately 100x higher affinity than VIP while both peptides have similar affinities for VPAC1-R and VPAC2-R. These receptors are widely distributed in the central and peripheral nervous system (Vaudry et al., 2000; Laburthe et al., 2007). The variable effects of PACAP are due to the activation of diverse signal transduction pathways and their outcomes depend on which receptor types have been activated. AC, PLC and Ca^2+^ are main effectors during the signal transduction mechanisms of PACAP (Spengler et al., 1993; Pisegna and Wank, 1996). PAC1R and VPAC1R are coupled to AC, which leads to cyclic adenosine 3′,5′-monophosphate (cAMP) level elevations and the subsequent activation of PKA, which in turn could activate the MAPK pathway. Both receptor types are coupled to PLC as well, which leads to the stimulation of Ca^2+^ mobilization and the activation of the protein kinase C (PKC) pathway. VPAC2R subtype also seems to activate the AC signaling pathway. Beyond the receptor types, activation of different pathways depends on the ligands, the tissue type, and the stage of the development (Filipsson et al., 1998; Basille et al., 2000; Vaudry et al., 2000).

PACAP and its receptors are present in the CNS and in peripheral organs of mammals (Arimura and Shioda, 1995; Vaudry et al., 2009). In the CNS it behaves as a neurotransmitter or neurotrophic factor and is expressed in the hippocampus, cerebellum, hypothalamus and in several brainstem nuclei (Hannibal, 2002; Lee and Seo, 2014). Several studies discussed its neuroprotective effects in neurodegenerative diseases such as in stroke, brain ischemic injuries, Alzheimer’s diseases and in Parkinsonism (Wang et al., 2008; Atlasz et al., 2010; Han et al., 2014; Matsumoto et al., 2016). Studies have revealed the expression of PAC1-R in the conjunctiva while PACAP/PAC1-R show higher expression in the lacrimal glands, in the cornea and in the retina (Wang et al., 1995; Elsas et al., 1996). In the retina, the nerve cell bodies in the GCL, some amacrine cells and horizontal cells show PACAP immunopositivity (Izumi et al., 2000; Denes et al., 2014). PAC1-R is strongly expressed in the GCL, in the INL and shows lower expression in the outer and inner plexiform layers (OPL, IPL) as well as in the ONL (Seki et al., 1997). To date, several studies have described the significant neuroprotective potential and neurotrophic effects of PACAP in relation to retinal metabolic disorders. Although its physiological action is incompletely elucidated, this peptide exerts neuroprotective and trophic actions by regulating cell survival and death, not only during the development and maturation of the nervous system but also in pathological conditions. Although pivotal roles in retinal metabolic disorders have been extensively investigated, the mechanisms are still not well understood and further signal transduction pathways may await to be revealed.

The primary aims of the present review are to summarize our knowledge about PACAP action in the retina in various physiological and pathological conditions (diabetic retinopathy, excitotoxic retinal injury and ischemic retinal lesion) and to discuss the potential signal transduction pathways in the context of its protective action. Particularly, we pay special attention to (i) the lack of PACAP in the retina and supplementation of PACAP during early postnatal development; (ii) PAC1-R subtypes in the retina and their possible involvement in the neuroprotective events; and (iii) role of PACAP in mobilizing the immune system, both white blood cells and chemical messengers, to achieve retinal neuroprotection. Finally, we summarize the synergistic and diverging pathways through which PACAP acts and achieves functional improvement in concerted action with other neuropeptides.

## PACAP Contra Retinal Degeneration With Metabolic Origins

As we mentioned above, the physiological role of PACAP in the adult retina is not well established. Clearly, an emerging theory is that the lack of endogenous PACAP would accelerate age-related degeneration (Reglodi et al., 2018). PACAP deficiency mimics aspects of age-related pathophysiological changes including increased neuronal vulnerability and systemic degeneration accompanied by increased apoptosis, oxidative stress, and inflammation thus mimicking early aging. In support of this theory, it has been proven recently that endogenous PACAP has a protective effect during retinal inflammation. Experiments with PACAP KO mice revealed that intraperitoneal injection of LPS induced markedly more seriously eye-inflammation in PACAP KO mice than in the wild type group. During the process of inflammation, protein kinase B (pAkt) and glycogen synthase kinase-3 (pGSK) levels decreased in PACAP KO mice while cytokines (sICAM-1, JE, TIMP-1) were elevated (Vaczy et al., 2018).

## Involvement of PACAP in Retinal Cell Development and Aging

In the CNS numerous extrinsic and intrinsic factors contribute to the formation of mature tissue by the precise regulation of the appropriate number and distribution of neurons. Neuropeptides influence many developmental processes of the CNS in a regulated way (Casini, 2005). In the developing retina, progenitor cells proliferate and differentiate into various retinal cell types as a result of numerous regulated cell cycle processes and develop into the final multi-layered structure of the retina. In postnatal (P6, P9) rat retinas PACAP treatment modulates cell death by activation of cAMP-PKA pathways (Silveira et al., 2002). Njaine et al. (2010) have investigated the exact timing and role of PACAP and its receptors in the cell generation of the developing rat retina. PAC1-R is expressed as early as E16 during development while VPAC1-R and VPAC2-R are expressed later, but then are present at all other stages. PACAP treatment resulted in an anti-proliferative effect by phosphorylation of CREB in cyclin D1 expressing retinal progenitor cells after PACAP receptor activation. Conversely, PACAP receptor activation led to a decreased level of cyclin D1 mRNA and further decreased by a combined treatment with PACAP and the cAMP degradation inhibitor IBMX. These findings have shown that PACAP has control over a subpopulation of progenitor cells and modulate cell proliferation in the developing retinal tissues (Njaine et al., 2010). Interestingly, PACAP shows both pro- and anti-apoptotic effects on postnatal retinal development in rat models. Caspase activity analysis has shown dose- and stage-dependent effects of PACAP on developmental apoptosis in rat retinas. Intravitreal injection of PACAP from postnatal day 1 (P1) to P7 induces apoptosis during the early stage of the retinogenesis. When 100 pmol PACAP was injected, it increased caspase 3/7 activity at P1, P3, and P5, but had no effect at P7. At P3, treatment repressed caspase 3/7 activity 18 h after the intravitreal injection, however, their levels increased 24 h post-injection. Apparently, PACAP treatment did not exert anti-apoptotic effects at P1, P5, and P7 rat retinas (Nyisztor et al., 2018). These findings warn us to re-evaluate PACAP action cautiously, always taking the timing and concentrations into account, especially in development. Unfortunately, not much is known about the functions of this peptide in mature retinas. Aging experienced as loss of function is accompanied by functional and morphological changes in retinal tissues (Gao and Hollyfield, 1992; Curcio and Drucker, 1993; Ramirez et al., 2001; Kovacs-Valasek et al., 2017). PACAP KO mice show accelerated age-related changes compared to wild type retinas. Altered structural changes included enhanced loss of ganglion cells and spouting of rod bipolar cell dendrites into the ONL in aging PACAP KO mice. Protein kinase C (PKC) α level in rod bipolar cells has been reduced in this condition. In contrast, GFAP levels have increased with an absence of endogenous PACAP. At the same time, PAC1-R has been upregulated in PACAP deficient young adult mice retinas. Surprisingly, the authors did not find differences in the histological structure of young adult PACAP KO and wild type mice (Kovacs-Valasek et al., 2017). These results suggest that PACAP contributes to maintaining the biochemical balance within neurons and glial cells. Thus, in the absence of this peptide, aging processes (e.g., reactive oxygen species formation) may gain strength earlier than in animals with normal PACAP levels.

## PACAP Receptor Types Expressed in Retina

In the retina, the presence of four PAC1-R isoforms has been verified during postnatal development. The Null isoform showed no impressive changes at early stages (P1 to P5), but then manifested a decline from P5 to P15. Null message levels fell almost to zero in early adult ages. The Hip isoform had a similar expression pattern. The Hiphop1 isoform showed one prominent peak at P10. The Hop1 splice variant did not change much between P1 and P5, but thereafter it showed a significant increase at P10, P15, and P20. This seems to be the major isoform during adult life. Depending on the type of the PAC1-R isoform, PACAP can induce precursor cells to exit the cell cycle (through activation of the Null isoform (Lu and DiCicco-Bloom, 1997) or can promote proliferation in neuroblasts (if they express the Hop isoform (Lu et al., 1998). Interestingly, expression of both Hip and Hop1 isoforms displays a sudden increase at P10 prior to eye opening. Due to technical difficulties, PAC1-R bearing retinal cells could not be sorted by their respective isoforms (isoform-specific antibodies are not available currently).

Based on these experimental results, a subsequent study has investigated the exact time period of isoform shift from postnatal day 5–10. The transcript level of Hip mRNA decreased from P6 through P9, while Hop1 expression level did not display any changes until P10. Consequently, a Hip/Hop1 isoform shift occurs between P6 and P8, which could alter PACAP functions in the postnatal rat retina (Denes et al., 2014). In contrast to the PAC1-R expression levels of the VPAC1-R receptor did not change during postnatal retinal development, though both the mRNA and protein could be detected in all selected time points. A similar scenario has been found in the case of VPAC2-R. Therefore, these receptors appear to be expressed in the newborn as well as in the adult retina, with similar intensity both at message and protein level (Lakk et al., 2012).

## Retinal Pathologies and PACAP

Retinal diseases fall into two main categories: inherited disorders and problems of metabolic origin. Both conditions have attracted substantial research interest. According to our PubMed survey, approximately 4,000 papers have been published in the last 10 years dealing with the former and about 3,000 with the latter. Approximately half of the papers deal either with diagnostic advances or treatment options. Below we shall summarize some of the experimental results regarding the three most common conditions: diabetic retinopathy, excitotoxic retinal injury and ischemic retinal conditions.

### Diabetic Retinopathy and PACAP

Diabetes is a multifactorial, metabolic disorder which appears to be the result of several pathological metabolic processes with increased morbidity statistics worldwide. In 2017, 425 million adults lived with diabetes and the size of the affected population will rise to 629 million until 2045 (International Diabetes Federation, 2017)^1^. DR is a microvascular complication of diabetes and the leading cause of vision loss (Cheung et al., 2010; Antonetti et al., 2012). DR is also considered as a chronic inflammatory disorder; low-grade inflammation has been observed in the retinas of both diabetic animals and human patients (Krady et al., 2005; Kern, 2007; Zeng et al., 2008). Patients with 1 type diabetes have a higher risk of DR than with the type 2 disease (Yau et al., 2012). DR has two distinguishable stages depending on the presence of neovascularization: an earlier non-proliferative phase characterized by abnormalities in the microvasculature, which could progress into a proliferative phase with macular neovascularization (Cheung et al., 2010).

The pathogenesis of DR includes increased polyol and hexosamine pathway activation, higher advanced glycation end-products production and the activation of PKC pathways. These altered signaling mechanisms could result in oxidative stress and chronic inflammation. Retinal microglial cells become activated and migrate in the subretinal space in several retinopathies, including DR (Zeng et al., 2000, 2008). The activation of microglia induced by hyperglycemia has been associated with the early development of DR, and occurs as early as electroretinographic modifications (Gaucher et al., 2007; Kern, 2007). Cytokines, released by activated microglia, were shown to contribute to neuronal cell death (Krady et al., 2005). They stimulate the production of cytotoxic substances, such as TNFα and ROS, proteases and even excitatory amino acids, which may induce neuronal degeneration. Leukocyte-mediated retinal cell apoptosis is among the earliest pathological manifestations of DR and results in the formation of a cellular-occluded capillaries, microaneurysms, and vascular basement membrane thickening (Kern and Engerman, 1995). Macrophages have long been known to play a major role in the pathogenesis of proliferative vitreoretinal disorders. In human DR, all types of macrophages could be detected regardless of clinical history and duration of the disease (Esser et al., 1993). Consequences of vascular occlusions contribute to neurodegeneration and dysfunction of the retina (Frank, 2004; Cheung et al., 2010; Giacco and Brownlee, 2010). Neuroprotective effects of PACAP in this pathological condition are complex because they have both structural, physiological and synaptic aspects as evidenced by many papers in this field (Table 1). In a rat model, intravitreal injection of PACAP ameliorated the structural changes of the retina in streptozotocin-induced DR. This treatment attenuates neuronal cell loss in the GCL, reduces cone cell degeneration and shows normal dopaminergic amacrine cell number compared to untreated diabetic retinas. These findings have demonstrated the significant neuroprotective effect of PACAP and its therapeutic potential in DR (Szabadfi et al., 2012). In their latest study, Maugeri et al. (2019) have provided evidence that PACAP1-38 protects not only neurons, but also the retinal pigmented epithelium both *in vivo* and *in vitro*. In another study, the intraocular PACAP injection attenuated the retinal injury by increasing the anti-apoptotic p-Akt, extracellular signal-regulated-kinase (p-ERK1/2), PKC and B-cell lymphoma 2 (Bcl-2) proteins levels, meanwhile the pro-apoptotic phosphorylated p38MAPK and activated caspase -3,-8, and -12 levels were decreased. As a result PACAP treatment significantly decreased apoptotic cell numbers compared to untreated diabetic rats and attracted a number of unidentified immune cells into the retina through the inner limiting membrane (Szabadfi et al., 2014). At the same time, electron microscopic analysis found altered synaptic structures in the diabetic retinas, in contrast to PACAP-treated diabetic groups, where more bipolar ribbon synapses appeared in the inner plexiform layer indicating higher levels of synapse-retention (Szabadfi et al., 2016). Giunta and his colleagues have described that MAPK transcripts levels were modified in the retina of diabetic rats during the early stages and the levels of PACAP, VIP and their receptors were all significantly downregulated as compared to non-diabetic rats (Giunta et al., 2012). At the same time, PACAP treatment has increased the PAC1-R expression in the retina, sometimes even in cells where PAC1-Rs are normally not present (Szabadfi et al., 2012).

**TABLE 1:** *In vivo* and *in vitro* experiments with PACAP application in DR (rat retina).

	**References**	**Study aim**	**Findings**
*In vivo*	Giunta et al., 2012	PACAP, VIP and their receptors expression change in retina of streptozotocin-induced diabetic rats.	The expression of peptides and their receptors were decreased after induction of diabetes. PACAP38 intravitreal injection restored diabetic changes in Bcl-2 and p53 expression to non-diabetic levels.
	Szabadfi et al., 2012	Highlights the protective effects of PACAP in diabetic retinopathy	PACAP ameliorated structural changes in DR, attenuated neuronal cell loss and increased the levels of PAC1-receptor and tyrosine-hydroxylase.
	D’Amico et al., 2015	The effects of PACAP in hyperglycemic retina is mediated by modulation of HIFs’ expression in retina.	In diabetic rats HIF-1α and HIF-2α expression decreased after PACAP intraocular administration while HIF-3α downregulated in retinas of STZ injected rats and increased after PACAP treatment.
	Szabadfi et al., 2016	Analyze the synaptic structure and proteins of PACAP-treated diabetic retinas after intravitreal PACAP administration.	In the PACAP-treated diabetic retinas more bipolar ribbon synapses were found intact in the inner plexiform layer than in DR animals. Degeneration of bipolar and ganglion cells could be ameliorated by PACAP treatment.
	D’Amico et al., 2017	Protective role of PACAP through IL1β and VEGF expression in rat diabetic retinopathy	PACAP reduced the IL-1β expression and downregulates VEGF, VEGFRs in STZ-treated animals.
	Maugeri et al., 2019	Effect of PACAP-38 against high glucose damage is mediated by EGFR phosphorylation in retina.	PACAP-38 induced p-EGFR over-expression in diabetic rats retina.
*In vitro*	Maugeri et al., 2019	Effect of PACAP-38 on ARPE-19 cells exposed to hyperglycemic/hypoxic insult	PACAP-38 treatment improved cell viability.

Unfortunately, there is no data available regarding VPAC1-R and VPAC2-R involvement in the PACAP response in the retina. However, VIP and PACAP have been shown to cooperate in functional studies by using other disease models (Schratzberger et al., 1998; Ganea and Delgado, 2003; Abad et al., 2016).

### Excitotoxic Retinal Injury and PACAP

Excitotoxic retinal injury in animal models mimics the changes associated with elevated intraocular pressure in human that causes glaucoma. Several studies have examined the neuroprotective effect of PACAP in excitotoxic retinal injuries. In normal conditions, glutamate is a neurotransmitter molecule in the retina, however, in high concentration it causes excessive stimulation of glutamate receptors and leads to excitotoxicity. In animal models of excitotoxic retinal injury, monosodium-glutamate treatment is used *in vivo* to model this pathological condition.

Monosodium glutamate (MSG) injection treatment has caused severe degenerations in neonatal rat retinas (Tamas et al., 2004; Atlasz et al., 2009). If prior to MSG treatment PACAP was injected unilaterally into the vitreous body of neonatal rat eyes, the MSG-induced degeneration became less pronounced. PACAP was applied in two different concentrations (1 and 100 pmol) to examine the dose-dependency of PACAP treatment in excitotoxic retinal injury. After MSG treatment the thickness of the entire retina was reduced by more than half and the reduction was especially due to the degeneration of the inner layers. Retinas of rats treated with 100 pmol PACAP showed significantly less damage than the retinas of animals treated with 1 pmol PACAP. These findings have described how PACAP could significantly attenuate the degeneration of the retina and underlined the importance of the dose-dependent effects of PACAP (Tamas et al., 2004). In another study, two different forms of PACAP (PACAP1-27, PACAP1-38) and their antagonists (PACAP6-38, PACAP6-27) have been tested in excitotoxic injury. The thickness of the retina has been significantly reduced, much of the IPL disappeared, the GCL and the INL cells intermingled and the ONL cells were swollen. During the investigation, PACAP1-38 and PACAP1-27 treated groups have shown retained retinal structure and the INL and GCL remained well separated. The two isoforms of PACAP have shown the same degree of neuroprotection after MSG treatments. The application of two PACAP antagonists after MSG injection did not ameliorate the MSG-induced retinal degenerations and led to a pronounced degeneration in the rat retina (Atlasz et al., 2009). During these experiments, the degenerations of the inner retinal layers were ameliorated by PACAP treatment. Note that PAC1-R distribution in the retina corresponds to the location of the protective effect because it shows the highest expression in the INL and in the GCL, and the lowest in the ONL and OPL (Seki et al., 2000). Another study examined the molecular background of signal transduction pathways underlying the neuroprotective effect of PACAP in MSG-induced retinal injury. The authors found that MSG inhibits the production of the anti-apoptotic molecules (phospho-PKA, phospho-Bad, Bcl-xL and 14-3-3 proteins) using rat models. PACAP treatment attenuates these effects by inducing the activation of the anti-apoptotic pathway by phosphorylation of PKA and Bad molecules and increasing the levels of Bcl-xL, and 14-3-3 proteins (Racz et al., 2007). These results highlighted that PACAP has a retinoprotective effect in glutamate induced injuries by reducing the pro-apoptotic pathways, while inducing anti-apoptotic signaling.

Interestingly, an enriched environment surrounding the experimental animals has also been shown to provide strong protective effect. A combination of enriched environment and PACAP treatment, however, did not further improve the protective effect, suggesting that these two treatments may utilize the same pathway for protection (Kiss et al., 2011).

### Retinal Ischemic Conditions and PACAP

Retinal ischemia, as well as ischemia-reperfusion, causes inflammation which leads to injury progression, though inflammation usually helps in neuronal repair. These conditions contribute to excess ROS production, increase intracellular calcium levels and initiate mitochondrial damage. In addition, MAPKs, nuclear factor κB (NFκB) and hypoxia-inducible factor 1α (HIF1α) are also activated when ischemic conditions elicit inflammation (Rayner et al., 2006; Wang et al., 2014; Kovacs et al., 2019). In the BCCAO model, PACAP activated one of the most important cytoprotective pathways, the PI3K-Akt, and suppressed the p38 MAPK and JNK pathways (Szabo et al., 2012) just like PARP inhibitors (Mester et al., 2009). Furthermore, a neurotrophic agent with a similar mode of action, CNTF, a member of the IL6 family (Wen et al., 2012), has also been tested in the form of intravitreal injection in preclinical studies. Using 12 animal models from 4 different species, researchers described a strong neuroprotective effect on photoreceptors and ganglion cells in the retina (Tao et al., 2002; Pease et al., 2009; Flachsbarth et al., 2014; Lipinski et al., 2015).

The effect of PACAP fragments has also been tested extensively in this model (Werling et al., 2014). The rationale for this study was that bioavailability and fast degradation of PACAP limit its therapeutic use and therefore scientific attention has been drawn to shorter fragments, especially the ones where C-terminus is truncated (Bourgault et al., 2009; Bourgault et al., 2011; Dejda et al., 2011). Therefore, it was necessary to test whether shorter PACAP fragments (4–13, 4–22, 6–10, 6–15, 11–15, and 20–31) have any effect on retinal lesions caused by chronic retinal hypoperfusion. Since the N-terminal fragments show a high similarity with the structure of VIP, and the 4-13 domain shows high selectivity to the PAC1-R, the prospect of creating a short and effective peptide fragment with a similar neuroprotective potential to PACAP seemed very promising. However, the authors came to the conclusion that the natural form of the peptide, PACAP1-38, is the most effective in retinal ischemia, and the 38 amino acid form of the peptide cannot be replaced by another fragment or another member of the peptide family (Werling et al., 2014). It has also been shown that PACAP mediates functional recovery after 14 days of intraocular treatment (Danyadi et al., 2014), probably through downregulation of VEGF production and glutamate release (D’Alessandro et al., 2014).

## Common, Synergistic and Diverging Pathways of PACAP Signaling to Achieve Functional Improvement

**FIGURE 1:**
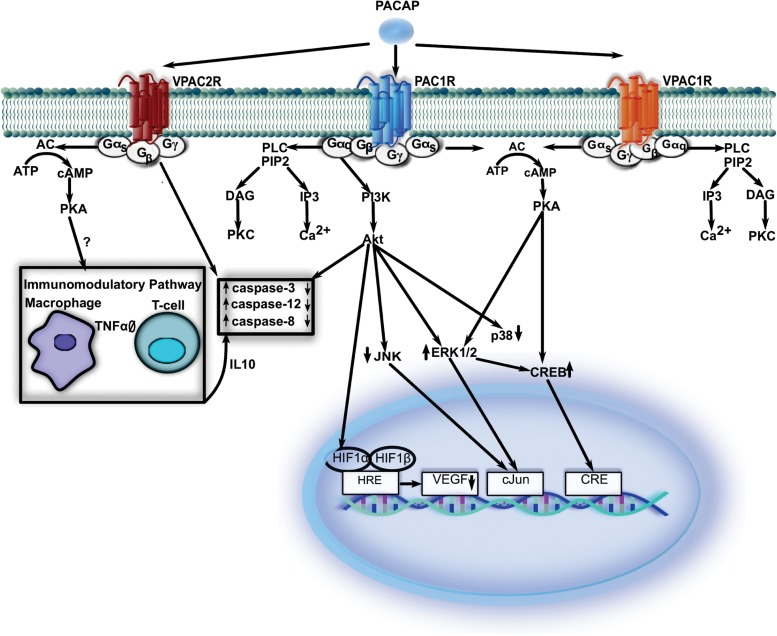
While PAC1-R-mediated signaling is at least partially synergistic with that of VPAC1-R, VPAC2-R that is inducible in some immune elements and utilizes additional signal molecules to restore normal function. VPAC1-R, vasoactive intestinal polypeptide receptor 1; VPAC2-R, vasoactive intestinal polypeptide receptor 2; PAC1-R, pituitary adenylate cyclase-activating polypeptide type I receptor; cAMP, cyclic adenosine3′,5′-monophosphate; AC, adenylate cyclase; PKA, protein kinase A; ATP, adenosine triphosphate; Gα/β/γ, G protein alpha/beta/gamma subunit; IL, interleukin; TNFα, tumor necrosis factor alpha; PLC, phospholipase C; PIP2, phosphatidylinositol 4,5-bisphosphate; DAG, diacylglycerol; IP3, inositol trisphosphate; PI3K, phoshoinositide 3-kinase; JNK, jun N-terminal protein kinase; ERK 1/2, extracellular signal-regulated-kinase 1/2; Akt, protein kinase; CREB, cAMP response element-binding protein; VEGF, vascular endothelial growth factor; HRE, hypoxia response element; HIF1α, hypoxia-inducible factor 1 α; HIF1β, hypoxia-inducible factor 1 β.

In the next few paragraphs, we aim to summarize the pathways activated, directly or indirectly by PACAP receptors (Figure 1). Unfortunately, most studies do not provide evidence which PACAP receptors are involved in the processes described below. Nevertheless, all the available data point to a critical function of PACAP in neuroprotection.

### Downregulation of Vascular Endothelial Growth Factor (VEGF)

Vascular endothelial growth factor, a dimeric glycoprotein functions as a mitogen by stimulating proliferation and migration of endothelial cells. It is also responsible for formation of new blood vessels (Ferrari and Scagliotti, 1996). The receptors of this signal molecule (VEGF- receptor 1, VEGF-R1 and VEGF- receptor 2, VEGF-R2) have tyrosine kinase domains and contribute to angiogenesis (Yancopoulos et al., 2000; Rahimi, 2006).

Among retinal cell types, mainly astrocytes, Müller glia cells, retinal pigment epithelium (RPE) and pericytes produce VEGF (Chalam et al., 2014). VEGF expression level is increased under low-oxygen concentrations through the induction of hypoxia-inducible factor 1 (HIF-1) expression. Hypoxia inducible factors (HIFs, see later) are modulators in hypoxia and cause endothelial cell transmigration across the RPE in the eye. These endothelial cells contribute to new vessel formation under VEGF control (Wang et al., 1995; Kaur et al., 2008; Skeie and Mullins, 2009). Elevated VEGF production leads to angiogenesis in order to supply tissues in hypoxic conditions (Kim et al., 2015). However, the newly generated blood vessels scatter light, and thus, instead of contributing to a better vision, they actually deteriorate visual acuity.

Studies have described diverse effects of PACAP on VEGF expression levels. Both PACAP and VIP are able to modulate HIF and VEGF expression during diabetic macular edema. VEGF expression is increased during hyperglycemic insult compared to control conditions. This effect can be ameliorated by PACAP or VIP treatment which could reduce the expression of VEGF and its receptors (Maugeri et al., 2017). Conversely, in another study, unrelated to diabetes, intravitreal treatment with PACAP has increased VEGF expression levels in rats after bilateral common carotid artery occlusion (Szabo et al., 2012). Although the results appear contradictory at first, at biological level the finding further demonstrates how profoundly protective PACAP is. In the extreme hypoxia at carotid artery occlusion the only survival strategy is more capillaries, that PACAP can also provide by an adaptive switch in its signaling bias. Nevertheless, the anti-VEGF effects of PACAP are clearly beneficial in patients suffering from DR conditions (Gabriel, 2013).

### Upregulation of HIF1alpha

HIFs are important transcriptional regulators under hypoxic circumstances targeting quite a few genes including VEGF (Hu et al., 2003). Under reduced oxygen conditions, these factors could modulate the cellular response to hypoxia (Loboda et al., 2010; Manalo et al., 2011). The HIF1 protein has two types of subunits (i.e., HIF1-α and HIF1-β) that show oxygen-dependent expression; while HIF1-β constitutively expressed, HIF1-α expression is increased under reduced oxygen concentrations (Jiang et al., 1996). During hypoxia, HIF1-α forms dimers with HIF1-β and the dimer binds to the HRE. This complex is able to regulate transcription of genes, which contribute to angiogenesis. One of them is the VEGF gene (Pugh and Ratcliffe, 2003). It has been previously shown that PACAP is able to modulate expression of HIFs in streptozotocin (STZ) induced diabetic retinas. After 3 weeks, HIF-1 alpha and HIF-2 alpha levels increased in diabetic groups and significantly decreased as a result of PACAP treatment. Conversely, HIF-3α was downregulated in diabetic rats and enhanced after intraocular administration of PACAP (D’Amico et al., 2015). In normal conditions, HIF1α level is reduced while HIF3α level increases, unlike in hypoxia or hyperglycemia, where their expression patterns are reversed. Treatments with VIP or PACAP reduce HIF1α levels and increase HIF3α levels in ARPE-19 cells under hyperglycemic conditions (Maugeri et al., 2017).

### Downregulation of c-Jun and p38 Kinases

c-Jun N-terminal protein kinase (JNK) and p38 kinase are members of the MAPK superfamily and they regulate apoptotic signaling pathways in cells (Estus et al., 1994; Ham et al., 1995; Mesner et al., 1995). JNK can have both pro- and anti-apoptotic effects (Ham et al., 1995; Xia et al., 1995; Lenczowski et al., 1997). In experiments using sodium arsenite (NaAsO_2__)_ to trigger neuronal apoptosis, both p38 kinase and JNK3 were upregulated and c-Jun phosphorylation was induced. The results showed that p38 kinase and JNK inhibitors attenuated apoptosis in cortical neurons and established the differences between JNK isoforms which differently contributed to the apoptotic processes (Namgung and Xia, 2000). It has also been described that intravitreal PACAP treatment decreased JNK, p38 activation and the activation of ERK1/2, AKT in hypoperfused rat retinas (Szabo et al., 2012). In MSG-induced retinal degeneration, PACAP treatment attenuated the activation of JNK and caspase 3 and increased the level of phospho-Bad (Racz et al., 2006). On the contrary, the same group demonstrated that PACAP treatment decreased the expression and activation of pro-apoptotic p38 in diabetic rat retinas (Szabadfi et al., 2014).

### Synergism With Other Peptidergic Mechanisms

The therapeutic potentials of different neuropeptides have been confirmed by numerous animal models of human diseases. These substances deserve prominent attention in the development of peptide-based therapeutic strategies of vision-threatening diseases.

The effectiveness of SST neuropeptide has been described in various pathological conditions of the retina. SST is an important neuromodulator and its immunoreactivity occurs mainly in the GABAergic amacrine cells in the retina (Feigenspan and Bormann, 1994; van Hagen et al., 2000). SST levels are downregulated at the early stage of DR (Carrasco et al., 2007). Topical administration of SST and its analogs have a preventive effect in retinal neurodegeneration in STZ-induced diabetes. It has been established that SST treatment inhibits extracellular glutamate accumulation, glial activation, ERG abnormalities and it modulates the proapoptotic/survival signaling pathways in experimental diabetes (Hernandez et al., 2013). Octreotide (OCTR) is a synthetic SST analog which, for example, in an ischemia/reperfusion injury study reduced cell loss, retinal thickness changes, ROS formation and inhibited NF-κB p65 activation. These findings demonstrated that OCTR application has a neuroprotective and antioxidant effect on ischemic injury in the retina (Wang et al., 2015). In another investigation, OCTR reduced hypoxia induced activation of STAT3 and HIF1 levels in retinal explants (Mei et al., 2012). OCTR and another SST analog (Woc4D) decreased neovascularization in the mouse model of oxygen-induced retinopathy (Higgins et al., 2002). A metabolomic analysis revealed the roles of PACAP, SP, and OCTR in *ex vivo* mouse models of retinal ischemia. These *ex vivo* results show a synergistic action of the above mentioned peptides. All treatments reduce VEGF overexpression, cell death and glutamate release and modulate pro-survival pathways by restoring IP3 signaling, cAMP levels and PIP2/PIP3 ratio in ischemia-induced retinal damages. It has also been demonstrated in ischemia related oxidative stress that PACAP and SP treatments help to cope with this condition and OCTR also contributes to the preventive effect in pathological processes (D’Alessandro et al., 2014).

Takuma et al. have investigated the effect of an enriched environment on memory impairments in PACAP deficient-mice. This environment ameliorates the memory impairments in knockout mice after 4 weeks and the beneficial effects of it were also observed if mice were returned to a standard environment after 2 weeks. The results showed that the levels of BDNF, phospho-ERK, phospho-CaMKII and *N*-methyl D-aspartate receptor subtype 2B (NR2B) in the hippocampus increased in an enriched environment and these factors are responsible for the ameliorating effect of the this environment on memory dysfunction. In PACAP −/− mice, however, these increased expression levels disappeared after 2 weeks when they were returned to standard housing, so in the lack of PACAP the long-lasting ameliorating effects of the enriched environment could not be verified (Takuma et al., 2014). An *in vitro* examination by Ogata and his colleagues have compared morphological effects of PACAP and BDNF on primary cultures of hippocampal neurons. Both PACAP and BDNF increased neurite length and numbers at a similar level, while PACAP increased the axon length only, but not the branching. Interestingly, the use of PACAP6-38 antagonist blocked both PACAP and BDNF-induced increases in axon length, suggesting that these two peptides may act through the same intracellular signal transduction machinery and that PACAP antagonists can interfere effectively with BDNF signaling (Ogata et al., 2015).

### Divergence in PACAP Receptor Signaling – How Immune Elements Are Recruited to Damaged Tissue Sites?

It has been demonstrated that immune cells express functional PACAP receptors. However, PAC1-R has minor roles in the immune response whereas VPAC1-R and VPAC2-R signaling evoke diverging effects. The former is constitutively expressed on macrophages, while the latter is inducible and particularly strongly effected by LPS (Abad et al., 2016). While VPAC1-R is thought to act mainly as an inhibitor of the immune response, VPAC2-R is able to accelerate inflammatory processes by initiating the production of several cytokines, most prominently IL-6 and IL-10. Additionally, D’Amico et al. (2017) have provided evidence that both IL1ß and VEGF levels are modified in diabetic rat retinas after PACAP administration. In peripheral organs PACAP also activates T-lymphocytes. In PACAP KO mice, however, PACAP treatment failed to reduce neutrophil infiltration into organs indicating that other indirect downstream PACAP signaling is also essential in this system (Martinez et al., 2005). VPAC1 and VPAC2 receptors, but not PAC1-R mRNA levels, were transiently induced in retinas 1 week following diabetes induction (Giunta et al., 2012). In the same diabetic condition, immune cells were attracted to the retina through the inner limiting membrane and resulted in strengthening of IL-6 but not tumor-necrosis factor (TNF) α-immunoreactivity in retinal ganglion cells. The reason for this difference is currently unknown and research is needed to clarify the underlying signaling routes. It is even more interesting that TNFα is dramatically increased in glaucoma and ischemia (Martinez et al., 2005). Therefore, it seems evident that not all of the microcircuitry-related disorders have identical immune cell recruitment pathways. This immune response may enhance the degeneration of the damaged cells. That, however, may be beneficial science when a protective signal like PACAP appears, it may be hasten the clearance of the dying elements, help to rearrange the neural connections and maintain the integrity of the remaining cells, to restore function as quickly as possible.

## Discussion

Our review highlights the importance of PACAP and, some other neuropeptides in retinal degenerative diseases with metabolic origins. Neuropeptides with their wide range of signaling potential could modulate the pathological pathways of retinal diseases through converging signal pathways. The question arises why these potentials are neglected in drug development and subsequent clinical trials. One of the difficulties of using natural peptides as protective agents is their relatively short half-life (in some cases it can be shorter than 1 min). The solution for this problem is to modify these peptides at their N and/or C termini in order to prevent degradation (acetylation, cyclization, N and/or C termini modification, PEGylation, D-amino acid substitution, etc.). In the case of PACAP, half-life can be longer than 4 h after some modifications (Mathur et al., 2016). Another potential problem using peptides as therapeutic agents is their limited passage through the blood brain barrier (Banks et al., 1993; Banks and Kastin, 1996). In the case of the retina there is no need for systemic administration since the peptides can injected into the vitreus body and must pass through the retinal inner limited membrane. Indeed it has been shown in the case of PACAP that it reaches the inner retinal layers after intravitreal injection (Werling et al., 2017).

At the same time, one of the mobilized downstream signals in the pathogenesis, VEGF is intensively targeted by different anti-VEGF therapies (Gabriel, 2013). While anti-VEGF therapies are expensive, synthesis and modification of peptides like PACAP are cost effective, so they may provide alternatives to the treatments available today in various retinal conditions, particularly in the case of DR. It would also be reasonable to consider the combination of modified neuropeptides, which can effectively counteract pathological retinal metabolic conditions. As discussed above, there are a number of candidates to be included in this mixture. In order to effectively protect every retinal cell type and layer we suggest trying the combination of modified BDNF, CNTF, OCTR, and PACAP. These substances together satisfy the following criteria (i) under normal conditions their native form is present in the retina in low concentration; (ii) each retinal cell type has a receptor for at least one of the four peptides; (iii) the signal transduction pathways behind the retinal receptors of these substances do not ameliorate or cross each other’s action; and (iv) none of them causes unwanted side effects even if they are given in higher concentrations. Considering that anti-VEGF drugs cost over 500 million pounds in Great Britain alone in 2015 (Hollingworth et al., 2017), alternatives are definitely needed, especially in low and medium income countries (Shanmugam, 2014). Clinical trials with the combinations of the above substances could be envisioned based on the results achieved on animal models in research laboratories.

## Author Contributions

All authors read and approved the final manuscript. RG wrote the manuscript and supervised the manuscript production. EP wrote the manuscript. VD gave expert advice and provided critical feedback.

## Conflict of Interest

The authors declare that the research was conducted in the absence of any commercial or financial relationships that could be construed as a potential conflict of interest.

## Footnotes

1 http://www.diabetesatlas.org

## Abbreviations

AC: adenylate cyclase
Akt: protein kinase B
ATP: adenosine triphosphate
BCCAO: bilateral carotid artery occlusion
Bcl-2: B-cell lymphoma 2 protein
BDNF: brain-derived neurotrophic factor
cAMP: cyclic adenosine 3 ′,5 ′ -monophosphate
CNS: central nervous system
CNTF: ciliary neurotrophic factor
CRE: cAMP response element
CREB: cAMP response element-binding protein
DAG: diacylglycerol
DR: diabetic retinopathy
ERK 1/2: extracellular signal-regulated-kinase 1/2
G α / β / γ: G protein alpha/beta/gamma subunit
GCL: ganglion cell layer
GFAP: glial fibrillary acidic protein
GSK: glycogen syntase kinase-3
HIF1 α: hypoxia-inducible factor 1 α
HRE: hypoxia response element
IL6: interleukin6
INL: inner nuclear layer
IP3: inositol trisphosphate
IPL: inner plexiform layer
JNK: jun N-terminal protein kinase
LPS: lipopolysaccharide
MAPK: mitogen activated protein kinase
MAPK: mitogen-activated protein kinase
MGS: monosodium glutamate
mRNA: messenger RNA
NaAsO2: sodium arsenite
NF κ B: nuclear factor κ B
NR2B: *N*-methyl D-aspartate receptor subtype 2B
OCTR: ocreotide
ONL: outer nuclear layer
OPL: outer plexiform layer
PAC1-R: pituitary adenylate cyclase-activating polypeptide type I receptor
PACAP: pituitary adenylate cyclase-activating polypeptide
PARP: poly ADP ribose polymerase
phospho-CaMKII: calcium/calmodulin-dependent protein kinase II
PI3K: phoshoinositide 3-kinase
PIP2: phosphatidylinositol 4,5-bisphosphate
PKA: protein kinase A
PKC α: protein kinase C alpha
PKC: protein kinase C
PLC: phospholipase C
PLC: phospholipase C
ROS: reactive oxygen species
RPE: retinal pigment epithelium
SST: somatostatin
STZ: streptozotocin
TNF α: tumor necrosis factor alpha
VEGF: vascular endothelial growth factor
VEGF-R: VEGF- receptor
VIP: mitogen activated protein kinase
VPAC1-R: vasoactive intestinal polypeptide receptor 1
VPAC2-R: vasoactive intestinal polypeptide receptor 2.
